# ChatGPT provides acceptable responses to patient questions regarding common shoulder pathology

**DOI:** 10.1177/17585732241283971

**Published:** 2024-09-25

**Authors:** Umar Ghilzai, Benjamin Fiedler, Abdullah Ghali, Aaron Singh, Benjamin Cass, Allan Young, Adil Shahzad Ahmed

**Affiliations:** 1 Baylor College of Medicine, Department of Orthopedic Surgery, Houston, TX, USA; 2 UT Health San Antonio, Department of Orthopaedics, San Antonio, TX, USA; 3 Sydney Shoulder Research Institute, Sydney Shoulder Specialists, Greenwich, New South Wales, Australia

**Keywords:** Artificial intelligence, machine learning, ChatGPT, shoulder, large language model

## Abstract

**Background:**

ChatGPT is rapidly becoming a source of medical knowledge for patients. This study aims to assess the completeness and accuracy of ChatGPT's answers to the most frequently asked patients’ questions about shoulder pathology.

**Methods:**

ChatGPT (version 3.5) was queried to produce the five most common shoulder pathologies: biceps tendonitis, rotator cuff tears, shoulder arthritis, shoulder dislocation and adhesive capsulitis. Subsequently, it generated the five most common patient questions regarding these pathologies and was queried to respond. Responses were evaluated by three shoulder and elbow fellowship-trained orthopedic surgeons with a mean of 9 years of independent practice, on Likert scales for accuracy (1–6) and completeness (rated 1–3).

**Results:**

For all questions, responses were deemed acceptable, rated at least “nearly all correct,” indicated by a score of 5 or greater for accuracy, and “adequately complete,” indicated by a minimum of 2 for completeness. The mean scores for accuracy and completeness, respectively, were 5.5 and 2.6 for rotator cuff tears, 5.8 and 2.7 for shoulder arthritis, 5.5 and 2.3 for shoulder dislocations, 5.1 and 2.4 for adhesive capsulitis, 5.8 and 2.9 for biceps tendonitis.

**Conclusion:**

ChatGPT provides both accurate and complete responses to the most common patients’ questions about shoulder pathology. These findings suggest that Large Language Models might play a role as a patient resource; however, patients should always verify online information with their physician.

**Level of Evidence:**

Level V Expert Opinion.

## Introduction

The information age has brought many resources for patients to learn about their illnesses. Shoulder pathology is no exception. Many patients turn to the Internet prior to consulting their physician; however, evidence suggests that, in general, online information is often suboptimal.^1,2^ Somerson et al.^3^ evaluated this for the shoulder, concluding that resources on the Internet were of mixed accuracy, quality and completeness. Further, they suggested that surgeons aim to direct patients towards higher quality resources.

Patients frequently query search engines regarding their shoulder pathology.^4^ However, newer technologies such as Large language models (LLMs), including ChatGPT (Open AI), are rapidly becoming sources of health information. LLMs have demonstrated some utility in patient explanation in many orthopedic subspecialties and other fields.^5–9^ With the ability to respond to patient queries in a human-like fashion, with vast quantities of information at its disposal, ChatGPT will probably play a large role in patient explanation and public health.

ChatGPT has shown promising results in medical education. Early iterations passed the United States Medical Licensing Examination battery of examinations.^10^ Additional testing demonstrated that ChatGPT (version 3.5) passed the Orthopaedic In-Training Examination (OITE) at the level of a postgraduate year 1 resident. The latest version, GPT-4, performed at the level of a passing fifth year resident, highlighting a rapidly improved level of competence between versions.^11^ Still, in its current form, Jain et al.^12^ concluded that ChatGPT's performance relied largely on outputting rote facts, and that its utility would be limited in orthopedic surgery. Further work showed that chatbot performance declined with question complexity, indicating deficiency in applying knowledge *versus* simply regurgitating facts.^13^ In the field of shoulder and elbow surgery specifically, Fiedler et al.^14^ noted that average performance by humans on the American Shoulder and Elbow Surgeons Maintenance of Certification Exam was superior to both ChatGPT 3.5 and 4, demonstrating that there remains a substantial knowledge gap between fellowship trained orthopedic surgeons and the chatbot. Additionally, the potential remains for the chatbot to produce misinformation or “hallucinate,” when the chatbot will fabricate information, presenting false information. This might represent a potential danger to patients, create pre-emptive false beliefs prior to the actual physician-patient encounter, and might be this resource's major limitation.^15–17^

Despite well-documented abilities on standardized tests, there is a dearth of literature assessing ChatGPT's ability to provide patients with specific information relating to shoulder pathology. This study aims to assess ChatGPT's ability to answer common patients’ queries regarding common shoulder pathologies and to evaluate the response for accuracy and completeness. We hypothesize that ChatGPT will produce both accurate and complete responses to common patient questions regarding shoulder pathology. These data might educate surgeons about the reliability of LLMs as patients’ information resources.

## Methods

ChatGPT (version 3.5) was queried in October of 2023 to identify the five most common shoulder pathologies. ChatGPT generated the following list of shoulder pathologies: biceps tendinitis, rotator cuff tears, shoulder arthritis, shoulder dislocation and adhesive capsulitis. It was then asked to generate the five most common patient questions regarding each of the above conditions. This produced 25 questions across all pathologies.

These 25 questions were then individually posed to ChatGPT and answers evaluated on Likert scales for both accuracy (1–6) and completeness (rated 1–3) as previously used for other assessments of the chatbot.^18,19^ For accuracy, a score of 1 indicated completely incorrect, 2 indicated more incorrect than correct, 3 indicated equal parts correct and incorrect, 4 indicated more correct than not, 5 indicated nearly all correct, and 6 indicated a completely correct answer. For completeness, a score of 1 indicated an incomplete response, 2 indicated an adequate response, with the minimum information required to be considered complete, 3 indicated a comprehensive response beyond what was expected. Responses rated at least “nearly all correct,” indicated by a score of 5 or greater for accuracy, and “adequately complete,” indicated by minimum of 2 for completeness, were considered to be sufficiently accurate and complete for patient education purposes.

The panel of three independent raters, consisting of fellowship-trained shoulder and elbow orthopedic surgeons, with a mean of nine years of independent practice, reviewed and scored all responses. Mean scores, for both accuracy and completeness, were calculated for each of the 25 questions. Additionally, mean scores for both metrics were calculated for all questions regarding each pathology. Inter-rater reliability was assessed for both metrics via intraclass correlation coefficient.

## Results

For all questions across all five conditions, ChatGPT's answers were deemed acceptable, producing answers rated at least “nearly all correct,” indicated by a score of 5 or greater for accuracy and “adequately complete,” indicated by a minimum of 2 for completeness. The average intraclass coefficient for accuracy scoring was 0.477 (95% CI: 0.050–0.742), suggesting subpar agreement among raters, while completeness scoring was 0.769 (95% CI: 0.470–0.899), demonstrating good reliability. As our scoring system was inherently subjective, some disagreement was anticipated.

**Figure 1.**
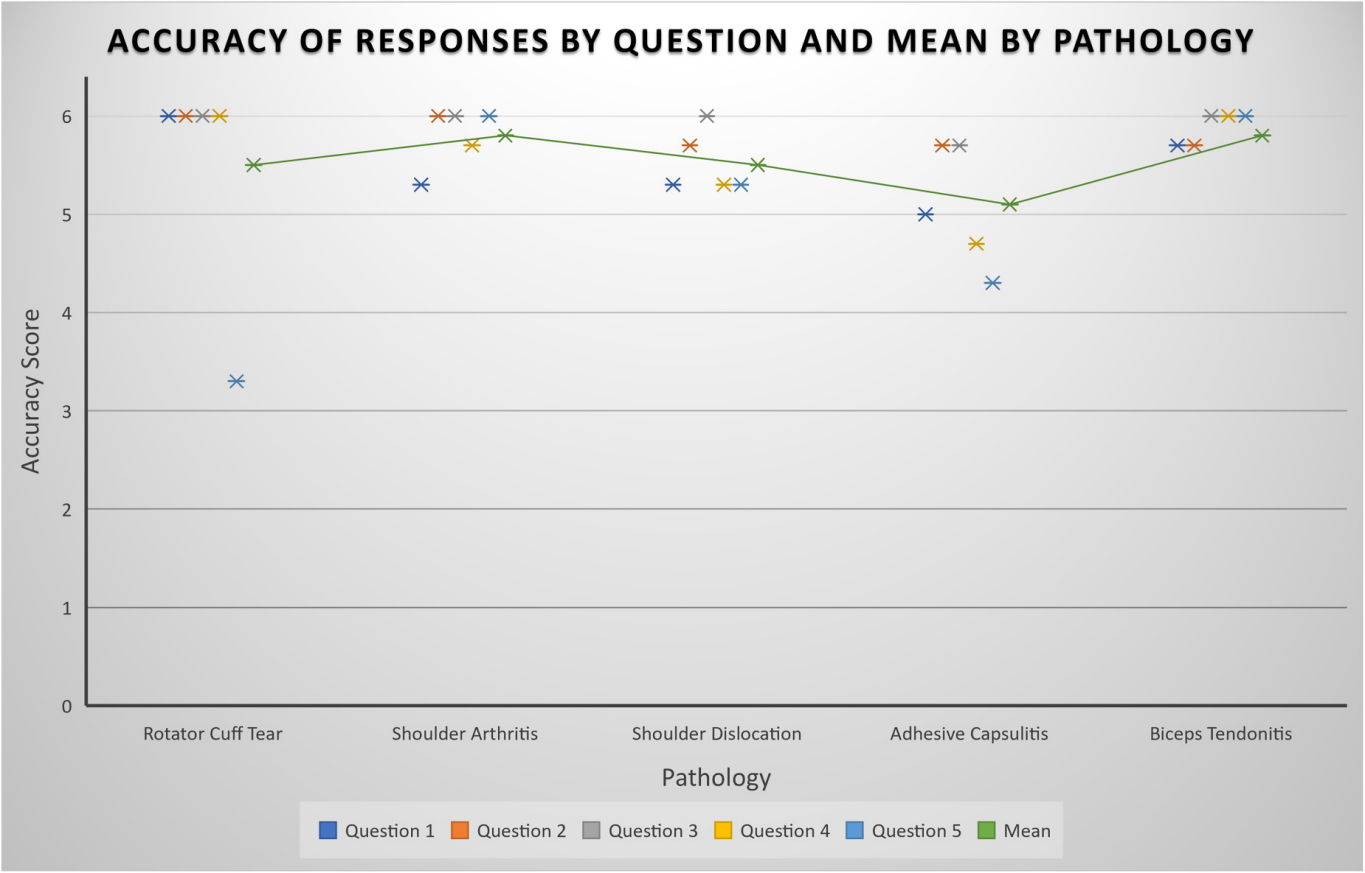
Plot representing the mean rating for accuracy of ChatGPT's responses by question. Mean accuracy rating per pathology is shown by the trendline.

**Figure 2.**
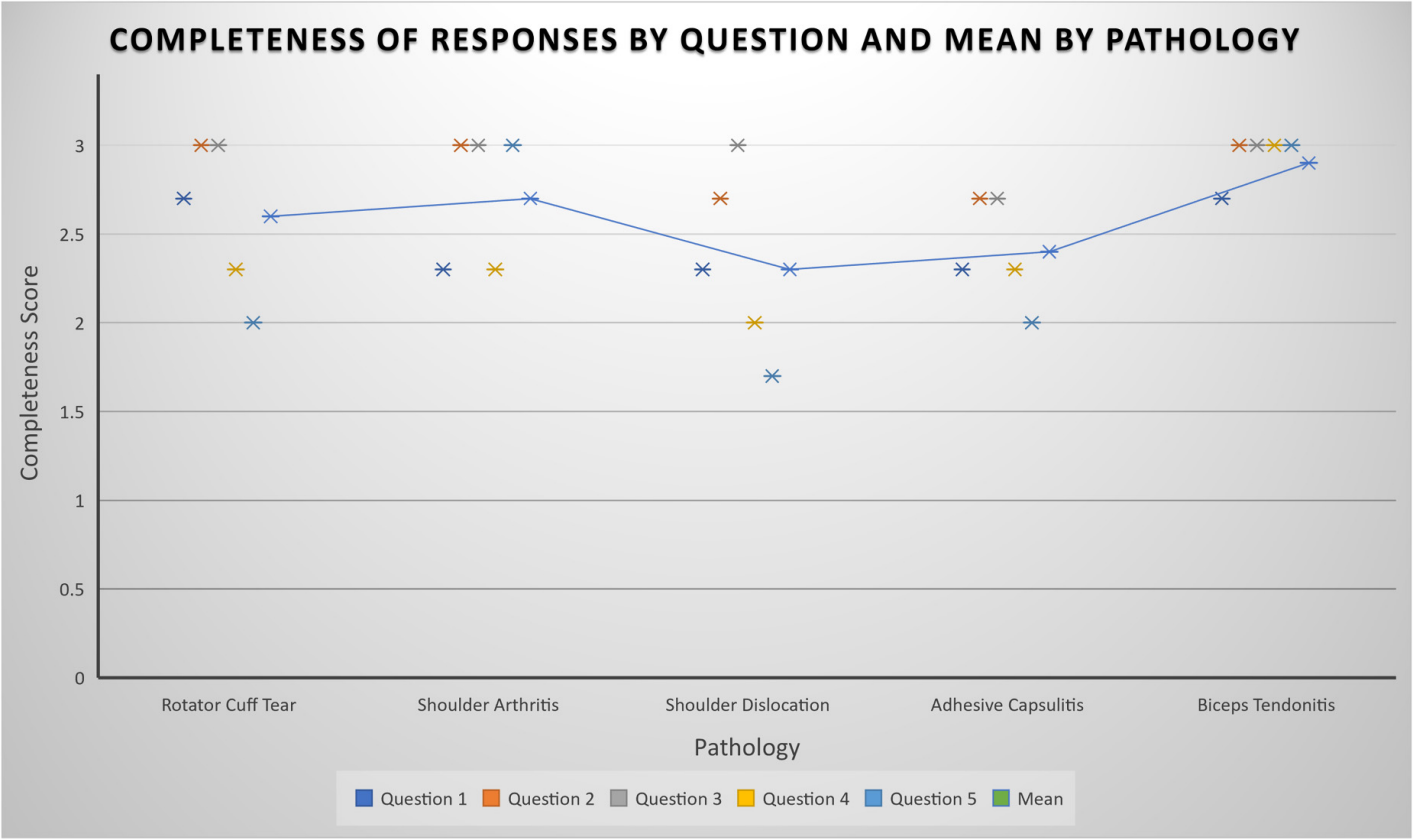
Plot representing the mean rating for completeness of ChatGPT's responses by question. Mean accuracy rating per pathology is shown by the trendline.

The mean scores for accuracy and completeness, respectively, were 5.5 and 2.6 for rotator cuff tears, 5.8 and 2.7 for shoulder arthritis, 5.5 and 2.3 for shoulder dislocations, 5.1 and 2.4 for adhesive capsulitis, 5.8 and 2.9 for biceps tendonitis. Full results for accuracy and completeness can be seen in Figures 1 and 2, respectively. ChatGPT generated questions and answers can be seen in full in Supplemental Material A to E.

## Discussion

This study assessed ChatGPT's ability to provide complete and accurate answers to patients’ questions regarding common shoulder pathology. For all five pathologies, the panel of three shoulder and elbow fellowship-trained orthopedic surgeons rated ChatGPT's answers at least “adequately complete” and “nearly all correct,” indicating sufficient accuracy and completeness, demonstrating acceptable performance in both domains. While peer-reviewed published literature and medical textbooks might provide higher quality information, these sources are often inaccessible to laypersons. The Internet and search engines provide available information but are challenging to parse through and assess for quality and reliability. ChatGPT might provide a solution to this dilemma, as its natural language processing abilities allow for human-like conversation, and vast reserve of training data enables it to synthesize evidence-based answers and deliver them in an accessible manner. While patients should still consult with physicians for questions about their health, our results show that LLMs might have a role in patient education.

These findings are corroborated by related literature. Multiple studies have reported that ChatGPT was able to provide patient information in shoulder surgery subspecialty specific areas, but noted that the reading level required was higher than American Medical Association and National Institute of Health recommended sixth grade reading level for patient materials.^20–22^ Furthermore, these studies noted a conspicuous absence of reliable citations, calling into question the reliability of these answers. Our own study reflected a similar phenomenon. While our queries were quite basic, the responses were generally lengthy and, while frequently technically correct, were perhaps overly detailed for simple patient questions. For example, ChatGPT proposed X-ray, ultrasound, magnetic resonance imaging, arthrogram, and computed tomography imaging for diagnosis of a rotator cuff tear. While all of these imaging modalities may be utilized at some stage of workup, and are valid options, it is unlikely a patient would require all of these for diagnosis.

These mixed findings are shared in other fields of orthopedic surgery. In the setting of elbow ulnar collateral ligament reconstruction, Johns et al.^23^ reported that ChatGPT was capable of producing satisfactory answers to common patient questions; however, they noted a large proportion of unsatisfactory responses, particularly as question complexity increased. In hand surgery, ChatGPT was able to provide medical information, at a layperson's level, on carpal tunnel syndrome management. However, it was noted the chatbot was prone to producing fabricated citations.^8^ In a study assessing responses to a variety of hand pathologies, Jagiella-Lodise et al.^24^ noted that the chatbot's responses were largely correct, but found them frequently incomplete. In the context of preoperative education prior to total hip arthroplasty, Mika et al.^7^ found that ChatGPT was able to provide largely satisfactory, largely evidence-based answers to most patients’ questions and was able to clarify answers when needed. They also noted that, when asked questions on controversial topics, the chatbot was seemingly unbiased. Similarly, in total knee arthroplasty, Magruder et al.^6^ found that ChatGPT was able to answer questions regarding clinical practice guidelines, albeit with lower scores in the domain of “consistency,” reflecting poor reliability. Vaira et al.^25^ drew similar conclusions in the context of head and neck surgery, reporting the chatbot's producing largely accurate information, with some incomplete answers, particularly as questions moved from closed-ended to more open-ended clinical scenarios.

Despite these promising reports in the literature, as well as our own, there are still a variety of issues with ChatGPT in healthcare. Goodman et al.^18^ noted that, while ChatGPT was able to provide highly accurate and complete answers to questions pertaining to a variety of medical specialties, there were notable outliers, and the chatbot would occasionally produce highly erroneous answers. They also noted that it was particularly concerning that these incorrect answers were delivered in the same conversational confident manner in which correct answers were given, suggesting a high potential for deception and misinformation. In our own study, the chatbot posited that rotator cuff tears may heal on their own. While not all tears require operative intervention, and many patients achieve functional and symptomatic improvement with conservative treatment, rotator cuff tears generally do not heal spontaneously. Many studies have reported on the “hallucination” phenomenon, which appears to be particularly common in citations for scientific articles.^26–28^ False statements, presented with fabricated, but very real-appearing references, present a problem for physicians, patients, and researchers. These “hallucinations” might result in misinformation, threaten patient safety by falsely conveying pre-conceived beliefs, and could cause problems in scientific work. Furthermore, it emphasizes the notion that patients should verify information from ChatGPT, and from any other sources, with their physician.

In a related study, Kuroiwa et al.^29^ noted a number of other concerns, including subpar reliability when assessing the chatbot's ability to aid patients in self-diagnosis. More disconcertingly, they reported that the chatbot did not sufficiently prompt the patient to seek medical attention, a potential risk to patient safety. The potential for this type of misuse, combined with the relatively early phase of the technology, is a challenge, and might, for now, preclude physicians’ recommendation of ChatGPT to patients. However, this problem is not unique to chatbots; the dangers of misdiagnosis with online tools predates ChatGPT.^30,31^ Simply stated, the adage to “consult with your physician” cannot be over-emphasized.

### Limitations

Although this study provided valuable and novel insights into the utility of an emerging technology, LLMs such as ChatGPT are not without limitation. The Likert-style questions, while commonly used in research, are ultimately a subjective measure of individual opinion. Our own analysis was somewhat limited by suboptimal interrater reliability, which is likely a reflection of this inherent subjectivity. Further work might attempt to create a validated objective scoring assessment of ChatGPT's responses to patient questions. Additionally, our analysis was limited to common questions regarding common pathologies. It is unclear how the chatbot would fare with less common pathologies and with more complex questions, as there is likely to be less training data on these topics.^32^ Our own study was limited to fairly basic questions, and no further lines of questioning were initiated to allow the chatbot to expound upon its response. Lastly, this study evaluated a specific iteration of the chatbot, ChatGPT version 3.5. More advanced versions, for example, the paid-access GPT-4, exist. These might produce different results. Furthermore, there are a variety of other online resources which patients might use for information, including other LLMs. Research comparing different LLM tools is warranted to avoid conflating a single company's technology with the larger field of LLMs across the board.

## Conclusion

ChatGPT was found to provide largely accurate and complete information to patients’ frequent questions about shoulder pathology. Mean response scores for all questions and pathologies were rated, at a minimum, nearly all correct and adequately complete, meeting our threshold for acceptable performance. However, there was subpar concordance among raters for accuracy ratings, but good agreement for completeness ratings. Initial results and the ease of interaction with ChatGPT demonstrate some possible utility in patient education. Still, despite their reliability, there remain many concerns regarding its use as a ubiquitous patient education tool. Surgeons should counsel patients on the importance of verifying, with their physician, information from ChatGPT or other online sources.

## Supplemental Material

sj-docx-1-sel-10.1177_17585732241283971 - Supplemental material for ChatGPT provides acceptable responses to patient questions regarding common shoulder pathology Supplemental material, sj-docx-1-sel-10.1177_17585732241283971 for ChatGPT provides acceptable responses to patient questions regarding common shoulder pathology by Umar Ghilzai, Benjamin Fiedler, Abdullah Ghali, Aaron Singh, Benjamin Cass, Allan Young and Adil Shahzad Ahmed in Shoulder & Elbow
