# Supplementary material for: ChatGPT provides acceptable responses to patient questions regarding common shoulder pathology
Source: Shoulder Elbow. 2024 Sep 25;17(5):625–30. doi: 10.1177/17585732241283971 (PMC11559869; doi:10.1177/17585732241283971)
Supplement: sj-docx-1-sel-10.1177_17585732241283971 - Supplemental material for ChatGPT provides acceptable responses to patient questions regarding common shoulder pathology [file sj-docx-1-sel-10.1177_17585732241283971.docx]

**Supplemental Tables**

**Supplemental Table A: Rotator Cuff Tear Questions and Answers**

| **Questions** | **Answers** |
| --- | --- |
| What is a rotator cuff tear? | A rotator cuff tear refers to a tear or damage in one or more of the tendons of the rotator cuff, which is a group of muscles and their corresponding tendons in the shoulder joint. These muscles and tendons play a crucial role in stabilizing and enabling the movement of the shoulder.   The rotator cuff is composed of four muscles: the supraspinatus, infraspinatus, teres minor, and subscapularis. These muscles originate from the scapula (shoulder blade) and attach to the humerus (upper arm bone). The tendons of these muscles form a cuff-like structure around the head of the humerus, helping to keep the shoulder joint stable and allowing for a wide range of shoulder movements.   A rotator cuff tear can occur in various ways, including acute injury (such as a fall or lifting a heavy object) or as a result of chronic wear and tear over time. Tears can range from partial thickness tears (involving only a portion of the tendon) to full-thickness tears (where the tendon is completely torn).   Common symptoms of a rotator cuff tear include:   Shoulder pain: Often felt on the outside of the shoulder and can worsen with arm movement.  Weakness: Difficulty in lifting or rotating the arm, particularly in activities that involve overhead motions.  Limited range of motion: Difficulty reaching overhead, behind the back, or reaching across the body.   It's important to note that not all rotator cuff tears cause significant pain or disability, and some may be managed without surgery through rehabilitation exercises, physical therapy, and other non-surgical treatments.   However, severe tears or tears causing significant functional impairment may require surgical repair to restore function and relieve pain. Treatment options will depend on the extent and type of the tear, as well as the individual's overall health and lifestyle. Always consult a healthcare professional for an accurate diagnosis and appropriate treatment plan. |
| What are the symptoms of a rotator cuff tear? | A rotator cuff tear can present various symptoms, which may vary in intensity based on the severity and location of the tear.   Common symptoms of a rotator cuff tear include:   Shoulder Pain: Persistent, aching pain in the shoulder, particularly on the outside or front of the shoulder. The pain may worsen when lifting or lowering the arm. Weakness: Weakened shoulder muscles, especially when attempting to lift or rotate the arm, particularly when performing actions like reaching or lifting objects.  Limited Range of Motion: Difficulty moving the shoulder and arm, especially with overhead activities or reaching behind the back.  Crepitus: A cracking, popping, or grating sensation or sound when moving the shoulder. Difficulty Sleeping: Discomfort or pain that interferes with sleeping, particularly when lying on the affected shoulder.  Pain at Rest: Pain that persists even when the shoulder is at rest, which may disturb daily activities or sleep.  Muscle Atrophy: Gradual loss of muscle mass (atrophy) in the affected shoulder due to decreased use and muscle weakness. Difficulty with Activities of Daily Living: Challenges with routine activities such as combing hair, reaching for objects, or getting dressed due to pain and limited mobility.  Pain Radiating Down the Arm: Pain that may radiate from the shoulder down the arm, possibly into the elbow.   It's important to note that some individuals with a rotator cuff tear may not experience noticeable symptoms, especially in the case of small tears or chronic tears that have developed gradually. Additionally, symptoms may worsen over time if the tear is not treated or managed appropriately. If you suspect a rotator cuff tear or are experiencing persistent shoulder pain or weakness, it's essential to consult a healthcare professional for an accurate diagnosis and appropriate treatment options, which may include physical therapy, medication, injections, or surgical repair, depending on the severity of the tear. |
| How is a rotator cuff tear diagnosed? | Diagnosing a rotator cuff tear typically involves a combination of a thorough medical history review, physical examination, and imaging studies. Here are the common diagnostic methods used to identify a rotator cuff tear:   Medical History and Physical Examination: The healthcare provider will begin by taking a detailed history, asking about symptoms, any recent injuries, previous shoulder issues, and overall health. They will then conduct a physical examination, evaluating the shoulder for signs of tenderness, weakness, range of motion, and other relevant factors. Clinical Tests: Various tests are used during the physical examination to assess the strength, stability, and function of the shoulder, such as the Jobe's test (empty can test), the drop arm test, the Hawkins-Kennedy impingement test, and the lift-off test. These help in detecting weakness and pain associated with rotator cuff tears.  Imaging Studies:  X-rays: Although they do not show soft tissues like tendons, X-rays can help rule out other issues such as arthritis or bone abnormalities. Ultrasound: This non-invasive imaging technique can provide real-time images of the rotator cuff and can help evaluate tears, their size, and location. MRI (Magnetic Resonance Imaging): MRI is commonly used to visualize the soft tissues of the shoulder, including the rotator cuff tendons. It can provide detailed images of the tear, its size, location, and severity. Arthrogram: An arthrogram involves injecting contrast dye into the shoulder joint before obtaining X-rays or an MRI. The dye helps highlight structures within the joint, making it easier to identify any tears or abnormalities. CT (Computed Tomography) Scan: CT scans may be used to assess bone structures and evaluate the extent of bone spurs or other bony abnormalities that may be contributing to the shoulder pain. Functional Tests: Functional tests may be used to assess the shoulder's overall function and capabilities, providing additional information to guide treatment decisions.  A combination of these diagnostic methods helps healthcare professionals accurately diagnose the presence, size, and location of a rotator cuff tear. The diagnosis is crucial in determining the appropriate treatment plan, which may include physical therapy, medication, injections, or surgical repair, based on the severity and nature of the tear. Always consult a healthcare professional for a proper evaluation and diagnosis. |
| What are the treatment options for a rotator cuff tear? | The treatment of a rotator cuff tear depends on various factors, including the size and severity of the tear, the patient's age, activity level, overall health, and the duration of symptoms. Treatment options for a rotator cuff tear may include both nonsurgical and surgical approaches:  Nonsurgical Treatment:  a. Rest and Activity Modification: Avoiding activities that aggravate the symptoms and allowing the shoulder to rest can help reduce pain and prevent further damage. b. Physical Therapy: Physical therapy focuses on strengthening the muscles around the shoulder to improve stability, flexibility, and range of motion. Therapists may also use modalities like heat, cold, or ultrasound for pain relief. c. Exercises: Specific exercises targeting the rotator cuff and shoulder muscles can help improve strength and function, aiding in the healing process and reducing pain. d. Pain Management: Over-the-counter pain medications (e.g., ibuprofen, acetaminophen) or prescribed pain relievers may help manage pain and inflammation. e. Corticosteroid Injections: Injections of corticosteroids into the shoulder joint can temporarily reduce inflammation and provide relief from pain.  Surgical Treatment:  a. Arthroscopic Repair: Arthroscopic surgery involves using small incisions and a camera (arthroscope) to guide the repair of the torn rotator cuff using sutures or anchors. b. Open Repair: Open surgery may be necessary for larger, more complex tears. It involves a larger incision to directly access and repair the torn rotator cuff. c. Tendon Transfer: In some cases, a tendon from another part of the body may be transferred to replace the torn portion of the rotator cuff. d. Debridement and Smoothing: This procedure involves removing frayed or damaged tissue and smoothing the edges of the remaining tendon to alleviate symptoms. e. Rehabilitation and Recovery: Following surgery, a structured rehabilitation program is crucial to restore strength, range of motion, and function of the shoulder. Physical therapy and exercises are an integral part of the recovery process.  The choice of treatment depends on factors like the size and location of the tear, the patient's age, activity level, and overall health. Nonsurgical approaches are often attempted first, particularly for smaller tears or when surgery may pose risks. Surgical intervention is considered when nonsurgical treatments fail to provide relief or for larger, more significant tears.  The ultimate decision regarding the treatment plan should be made in consultation with an orthopedic surgeon or a healthcare professional specializing in shoulder injuries. |
| Can a rotator cuff tear heal on its own without surgery? | Yes, a rotator cuff tear can potentially heal on its own without surgery, especially in the case of smaller tears or partial thickness tears. The ability of a tear to heal without surgery largely depends on several factors, including the size and location of the tear, the overall health and age of the individual, and their level of physical activity.  Here are some factors that influence whether a rotator cuff tear can heal without surgery:  Tear Size and Location: Smaller tears, particularly partial tears, have a higher likelihood of healing without surgical intervention compared to larger, full-thickness tears. Acuteness of the Injury: Acute tears resulting from recent injuries may have a better chance of healing compared to chronic tears that have developed gradually over time. Age and Overall Health: Younger individuals generally have a better healing capacity, as do those with good overall health and no underlying medical conditions that impede the healing process. Activity Level and Lifestyle: Adjusting activity levels and avoiding movements that aggravate the tear can facilitate healing. Physical therapy and specific exercises to strengthen the rotator cuff muscles may also aid in natural healing. Conservative Treatments: Nonsurgical treatments such as rest, activity modification, physical therapy, and appropriate pain management can support the healing process and improve symptoms associated with a rotator cuff tear.  It's essential to work closely with a healthcare professional, usually an orthopedic specialist or physical therapist, to determine the most suitable treatment plan based on the individual circumstances. Nonsurgical approaches are often the first line of treatment and may include a combination of rest, exercises, pain management, and physical therapy to help rehabilitate the shoulder and promote healing.  Regular follow-up evaluations and imaging studies (e.g., MRI) may be conducted to monitor the progress of the healing process and adjust the treatment plan as needed. If nonsurgical approaches are ineffective or if the tear worsens, surgical intervention may be considered as a later option. Always consult a healthcare professional for personalized advice and guidance regarding the management of a rotator cuff tear. |

**Supplemental Table B: Shoulder Arthritis Questions and Answers**

| **Questions** | **Answers** |
| --- | --- |
| What is shoulder arthritis, and what causes it? | Shoulder arthritis is a condition characterized by the inflammation, degeneration, and wearing down of the shoulder joint. The shoulder joint is a ball-and-socket joint that connects the upper arm bone (humerus) to the shoulder blade (scapula). Arthritis in the shoulder can affect the smooth gliding and movement of the joint, leading to pain, stiffness, and reduced range of motion.  There are several types of arthritis that can affect the shoulder:  Osteoarthritis (Degenerative Arthritis): Osteoarthritis is the most common type of shoulder arthritis. It occurs due to the gradual wear and tear of the cartilage in the joint over time. As the cartilage breaks down, the bones may rub against each other, causing pain and reduced function. Rheumatoid Arthritis: Rheumatoid arthritis is an autoimmune disorder in which the body's immune system mistakenly attacks the lining of the joint (synovium), leading to inflammation, pain, and eventual damage to the joint. Post-Traumatic Arthritis: Post-traumatic arthritis can develop after a severe shoulder injury, such as a fracture or dislocation. The trauma to the shoulder can damage the cartilage and accelerate the onset of arthritis. Avascular Necrosis (Osteonecrosis): Avascular necrosis occurs when the blood supply to the bone in the shoulder is disrupted, leading to bone death and subsequent arthritis. Rotator Cuff Tear Arthropathy: Rotator cuff tear arthropathy occurs when a severe, long-standing rotator cuff tear leads to arthritis in the shoulder joint due to altered mechanics and instability.  Risk factors that may contribute to the development of shoulder arthritis include:  Aging: The risk of shoulder arthritis increases with age. Previous shoulder injuries: Prior injuries or trauma to the shoulder can predispose an individual to arthritis. Overuse or repetitive strain: Certain occupations or sports that involve repetitive overhead movements may contribute to arthritis. Genetics: Some individuals may have a genetic predisposition to developing arthritis. |
| What are the symptoms of shoulder arthritis? | Shoulder arthritis is a condition that involves inflammation, pain, and stiffness in the shoulder joint due to the degeneration of cartilage. The symptoms of shoulder arthritis can vary in intensity and may worsen over time.   Common symptoms include:  Pain: Persistent pain in the shoulder joint, typically worsening with movement and activity. The pain may range from a dull ache to sharp, shooting sensations. Stiffness: Difficulty moving the shoulder, especially in the morning or after periods of inactivity. Stiffness may improve with gentle movement and activity. Limited Range of Motion: Reduced ability to move the shoulder through its full range of motion, making it challenging to lift the arm or perform overhead activities. Weakness: Weakness in the shoulder muscles, leading to difficulty lifting objects or performing daily tasks that require arm strength. Crepitus: A grinding, crackling, or popping sensation or noise within the shoulder joint during movement. Swelling and Tenderness: Swelling and tenderness in and around the shoulder joint, often accompanied by warmth and redness. Difficulty Sleeping: Pain and discomfort that interfere with sleep, especially when lying on the affected shoulder. Muscle Atrophy: Gradual loss of muscle mass (atrophy) in the affected shoulder due to decreased use and muscle weakness. Fatigue: Feeling fatigued or tired due to the effort required to move the shoulder and manage the associated discomfort. Difficulty with Activities of Daily Living: Challenges with routine activities, such as reaching for objects, combing hair, or putting on clothes due to pain and limited mobility. Radiating Pain: Pain that may radiate from the shoulder down the arm, possibly into the elbow or upper back. |
| How is shoulder arthritis diagnosed? | The diagnosis of shoulder arthritis typically involves a combination of a thorough medical history review, physical examination, and various imaging studies. Here's how shoulder arthritis is commonly diagnosed:  Medical History: The healthcare provider will begin by taking a detailed history, asking about symptoms, their duration, intensity, any previous injuries, medical conditions, or treatments related to the shoulder. Physical Examination: The provider will conduct a physical examination of the shoulder, evaluating for signs of pain, tenderness, range of motion limitations, muscle strength, and any joint deformities or abnormalities. X-rays: X-rays are usually the first imaging test performed. They can reveal joint space narrowing, bone spurs, and other degenerative changes associated with arthritis. Magnetic Resonance Imaging (MRI): An MRI may be recommended to provide detailed images of the soft tissues in and around the shoulder joint, helping to assess the condition of the cartilage, ligaments, tendons, and other structures. CT (Computed Tomography) Scan: CT scans can provide detailed images of the bones in the shoulder and may be used to assess the degree of bone degeneration, bone spurs, and the overall condition of the joint. Ultrasound: Ultrasound may be used to evaluate soft tissue structures, such as tendons and muscles, and assess for any inflammation or abnormalities. Arthrogram: In some cases, a shoulder arthrogram may be performed. This involves injecting a contrast dye into the shoulder joint before obtaining X-rays or an MRI. The dye helps highlight structures within the joint, making it easier to identify abnormalities. Blood Tests: While there's no specific blood test to diagnose shoulder arthritis, certain blood tests may be done to rule out other possible causes of joint pain and inflammation, such as rheumatoid arthritis. Bone Scan: A bone scan may be performed in specific cases to assess the overall bone health and identify areas of increased bone activity.  By combining information from these diagnostic methods, healthcare professionals can accurately diagnose shoulder arthritis, determine its severity, and develop an appropriate treatment plan. The goal is to tailor the treatment to the individual's specific condition, considering factors like the type and extent of arthritis, overall health, lifestyle, and desired level of activity. Always consult a healthcare professional for personalized evaluation and guidance. |
| What are the treatment options for shoulder arthritis? | The treatment of shoulder arthritis aims to alleviate pain, improve function, and enhance the individual's quality of life. The approach to treatment may vary based on the type and severity of the arthritis, the individual's age, overall health, activity level, and personal preferences. Here are common treatment options for shoulder arthritis:  Non-Surgical Treatments:  a. Pain Medication: Over-the-counter nonsteroidal anti-inflammatory drugs (NSAIDs) or acetaminophen may be used to manage pain and reduce inflammation. b. Physical Therapy: A structured physical therapy program can help improve shoulder strength, flexibility, and range of motion. Therapists may incorporate exercises and stretches specific to shoulder arthritis. c. Occupational Therapy: Occupational therapists can teach adaptive techniques and assistive devices to help with daily activities and reduce strain on the shoulder joint. d. Activity Modification: Avoiding activities that exacerbate shoulder pain and modifying movements to reduce stress on the joint can help manage symptoms. e. Corticosteroid Injections: Corticosteroid injections directly into the shoulder joint can provide temporary relief from pain and inflammation. f. Viscosupplementation: Hyaluronic acid injections may be used to lubricate and cushion the joint, providing pain relief for some individuals. g. Hot or Cold Therapy: Applying heat or cold packs to the shoulder may help alleviate pain and stiffness.  Lifestyle Modifications: Maintaining a healthy weight and adopting a well-balanced diet can help reduce stress on the shoulder joint and improve overall joint health.  Assistive Devices: Supports like slings or braces may provide stability and reduce stress on the shoulder joint.  Surgical Treatments:  a. Arthroscopy: Arthroscopic surgery may be performed to remove loose, damaged tissue, bone spurs, or inflamed synovial lining to alleviate symptoms. b. Shoulder Resurfacing: This procedure involves replacing the damaged portion of the humeral head (upper arm bone) with a metal cap. c. Shoulder Replacement (Total Shoulder Arthroplasty): In severe cases, a shoulder replacement surgery may be recommended, involving replacing the damaged shoulder joint with a prosthetic implant. d. Reverse Total Shoulder Arthroplasty: This procedure is an option for individuals with severe arthritis and a rotator cuff tear. It involves reversing the ball-and-socket configuration to improve stability and function. e. Hemiarthroplasty: In this surgery, only the humeral head is replaced with a prosthetic implant.  The choice of treatment depends on the severity of the arthritis, the individual's overall health, lifestyle, and goals. The ultimate treatment plan is usually tailored to each individual's specific circumstances. It's essential to work closely with a healthcare professional to determine the most suitable treatment approach and achieve the best outcome. |
| Can shoulder arthritis be managed without surgery? | Yes, shoulder arthritis can often be managed effectively without surgery, especially in the early stages or when the arthritis is not severely impacting daily activities. Non-surgical treatments can help alleviate pain, improve shoulder function, and enhance the individual's quality of life.   Here are non-surgical management options for shoulder arthritis:  Pain Medication: Over-the-counter nonsteroidal anti-inflammatory drugs (NSAIDs) or acetaminophen can help manage pain and reduce inflammation. Physical Therapy: A structured physical therapy program can be highly beneficial. Physical therapists can design exercises to improve shoulder strength, flexibility, and range of motion, ultimately enhancing function and reducing pain. Occupational Therapy: Occupational therapists can provide strategies and techniques to adapt daily activities and reduce strain on the shoulder joint. Activity Modification: Avoiding or modifying activities that exacerbate shoulder pain and utilizing proper techniques and ergonomics can help manage symptoms. Corticosteroid Injections: Corticosteroid injections directly into the shoulder joint can provide temporary relief from pain and inflammation, often lasting several months. Viscosupplementation: Hyaluronic acid injections may be used to lubricate and cushion the joint, providing pain relief for some individuals. Hot or Cold Therapy: Applying heat or cold packs to the shoulder may help alleviate pain and stiffness. Assistive Devices: Supports like slings or braces may provide stability and reduce stress on the shoulder joint. Lifestyle Modifications: Maintaining a healthy weight and adopting a well-balanced diet can help reduce stress on the shoulder joint and improve overall joint health. Regular Exercise: Low-impact exercises like swimming, stationary biking, and gentle stretching can help maintain shoulder mobility and strengthen supporting muscles without causing further damage. Education and Self-Care: Learning about shoulder arthritis and its management, following prescribed exercises, maintaining good posture, and incorporating self-care techniques can be valuable in managing symptoms.  While surgery may be considered for severe cases or when non-surgical treatments are ineffective, many individuals experience relief and improved shoulder function through non-surgical approaches. It's important to consult with a healthcare professional to develop a personalized treatment plan based on individual circumstances and the progression of the condition. |

**Supplemental Table C: Shoulder Dislocation Questions and Answers**

| **Questions** | **Answers** |
| --- | --- |
| What is a shoulder dislocation, and how does it happen? | A shoulder dislocation occurs when the ball-shaped head of the upper arm bone (humerus) comes out of the shallow socket (glenoid) of the shoulder blade (scapula). This displacement disrupts the normal alignment of the shoulder joint, causing significant pain, instability, and limited mobility.  Shoulder dislocations typically occur due to a sudden and forceful movement or trauma to the shoulder. Common ways a shoulder dislocation can happen include:  Traumatic Injury: Most shoulder dislocations result from a traumatic event, such as a fall onto an outstretched arm, a direct blow to the shoulder, or a sudden twisting or rotational force applied to the shoulder joint during sports or accidents. Sports Injuries: Activities like contact sports (e.g., football, rugby), skiing, basketball, and gymnastics carry a higher risk of shoulder dislocations due to the potential for falls, collisions, or awkward landings. Accidental Falls: A fall, especially onto an outstretched hand or arm, can cause the shoulder to dislocate as the impact forces the arm out of its socket. Motor Vehicle Accidents: High-impact accidents, particularly those involving rapid deceleration or collision forces, can lead to shoulder dislocations. Seizures or Electric Shocks: Severe muscle contractions during seizures or electric shocks may forcefully dislocate the shoulder. Repetitive Strain or Overuse: Chronic overuse, repetitive stress, or improper shoulder mechanics over time can weaken the supporting structures of the shoulder, making it more susceptible to dislocation.  It's important to note that some individuals may have a predisposition to shoulder instability or a history of dislocations, making them more susceptible to recurrent shoulder dislocations with less force or trauma.  When a shoulder dislocation occurs, the ligaments, tendons, and other supporting structures around the shoulder may stretch or tear, contributing to the dislocation. It's crucial to seek prompt medical attention to reduce and stabilize the shoulder joint, which often involves a healthcare professional carefully maneuvering the arm back into its socket (reduction). After reduction, appropriate treatment, immobilization, rehabilitation, and preventive measures are essential to manage the injury and reduce the risk of future dislocations. |
| What are the symptoms of a shoulder dislocation? | A shoulder dislocation is a significant injury that often results in immediate and noticeable symptoms.   Common symptoms of a shoulder dislocation include:  Intense Shoulder Pain: Sudden, severe pain in the shoulder area is a hallmark symptom. The pain may worsen with movement or attempted use of the arm. Obvious Deformity or Abnormal Appearance: The affected shoulder may appear visibly out of place, disfigured, or deformed due to the displacement of the shoulder joint. Inability to Move the Arm: Limited or complete loss of movement in the shoulder and arm due to the dislocation, making it difficult to perform even simple tasks. Swelling and Bruising: Swelling and bruising around the shoulder and upper arm are common. The area may appear swollen, and the skin may show signs of bruising or discoloration. Instability and Weakness: A sense of instability or looseness in the shoulder, making it feel like it may pop out again. Associated weakness in the affected arm may be present. Numbness or Tingling: Numbness, tingling, or a "pins and needles" sensation may occur in the arm or hand due to pressure on nerves or blood vessels during the dislocation. Muscle Spasms: Muscle spasms may occur around the shoulder as the body attempts to protect the injured area. Difficulty in Breathing or Swallowing: In severe cases, the dislocation may cause pressure on surrounding structures, leading to difficulty breathing or swallowing.  It's important to seek immediate medical attention if a shoulder dislocation is suspected. Delaying treatment may increase the risk of complications and hinder the successful reduction (putting the shoulder back into place) of the dislocated joint. Healthcare professionals, typically in an emergency room or urgent care setting, can provide the appropriate care, including reduction and subsequent evaluation to determine the extent of the injury and formulate a suitable treatment plan. |
| How is a shoulder dislocation diagnosed? | Diagnosing a shoulder dislocation typically involves a combination of a thorough medical history review, physical examination, and imaging studies. Here's how a shoulder dislocation is commonly diagnosed:  Medical History: The healthcare provider will begin by taking a detailed history, asking about the circumstances of the injury, symptoms, previous shoulder dislocations, any associated pain, and past medical conditions related to the shoulder. Physical Examination: A physical examination is crucial to assess the affected shoulder. The provider will carefully examine the shoulder for signs of deformity, swelling, tenderness, instability, and assess the range of motion, muscle strength, and sensation in the arm. X-rays: X-rays are often the first imaging tests performed to confirm the diagnosis and evaluate the extent of the dislocation. X-rays can show the position of the displaced bone and any associated fractures. Magnetic Resonance Imaging (MRI): In some cases, an MRI may be performed to assess soft tissues, ligaments, tendons, and detect any injuries that may not be visible on X-rays. CT (Computed Tomography) Scan: A CT scan may be used to provide detailed images of the shoulder joint and surrounding structures, particularly in complex or severe dislocations. Ultrasound: Ultrasound may be used to assess soft tissue structures and provide real-time imaging of the shoulder. Clinical Tests: Various tests and maneuvers may be performed during the physical examination to assess shoulder stability, muscle strength, and the likelihood of a dislocation. Comparison with the Unaffected Shoulder:  Comparing the affected shoulder with the unaffected shoulder can help the healthcare provider identify differences and abnormalities.  History of Previous Dislocations: If the individual has a history of previous shoulder dislocations, this information is considered during the diagnostic evaluation.  Based on the results of these diagnostic methods, the healthcare provider can confirm the diagnosis of a shoulder dislocation, determine the type and severity of the dislocation, identify any associated injuries or fractures, and develop an appropriate treatment plan. Early diagnosis and appropriate treatment are essential for the successful management and recovery of a shoulder dislocation. Always consult a healthcare professional for personalized evaluation and guidance. |
| What are the treatment options for a shoulder dislocation? | The treatment of a shoulder dislocation aims to reduce the dislocation, relieve pain, stabilize the joint, prevent further dislocations, and rehabilitate the shoulder to restore its function.   Treatment options for a shoulder dislocation may include both immediate and follow-up interventions:  Immediate Care (Reduction):  Closed Reduction: This involves manually maneuvering the dislocated shoulder bone (humerus) back into its normal position (reduction). It's typically performed by a healthcare professional in an emergency room or clinic using specific techniques to realign the joint. Immobilization: After reduction, the shoulder is often immobilized using a sling or a shoulder immobilizer to restrict movement and allow the ligaments and tissues to heal. Pain Management: Pain relievers, ice packs, and anti-inflammatory medications may be used to manage pain and reduce inflammation. Physical Therapy: Physical therapy is essential for rehabilitation. It includes exercises to strengthen the shoulder muscles, improve range of motion, and stabilize the joint. Gradually, functional exercises are introduced to regain strength and movement.  Surgical Intervention: Surgery may be considered in certain cases:  Recurrent Dislocations: If a person experiences repeated shoulder dislocations or has a high risk of recurrence, surgical procedures to stabilize the shoulder joint (e.g., Bankart repair, capsulorrhaphy) may be recommended. Fractures or Other Injuries: Surgery may be necessary if the dislocation is associated with fractures or severe soft tissue injuries. Rehabilitation and Recovery: A structured rehabilitation program is crucial to restore shoulder strength, range of motion, and function. Physical therapists guide the recovery process, ensuring a gradual return to daily activities, work, and sports.  Preventive Measures: Once the initial treatment is completed, individuals are advised to follow preventive measures such as strengthening exercises, maintaining good posture, and avoiding risky activities to reduce the risk of future dislocations. Lifestyle Modifications: Adjustments in activities and sports participation may be recommended to prevent re-injury or recurrence of dislocations.  The choice of treatment depends on various factors, including the severity of the dislocation, the individual's age, activity level, occupation, and overall health. Treatment plans are tailored to the specific needs and circumstances of each person. It's crucial to work closely with a healthcare team to determine the most suitable treatment approach and optimize the recovery process. |
| Can a shoulder dislocation happen more than once, and how can it be prevented? | Yes, a shoulder dislocation can happen more than once, especially if an individual has a history of previous dislocations or if the shoulder has been weakened or stretched due to repeated injuries. Recurrent shoulder dislocations can be a concern and often require preventive measures and, in some cases, surgical intervention to stabilize the shoulder joint.  Prevention of recurrent shoulder dislocations involves various strategies:  Strengthening Exercises: Performing regular shoulder-strengthening exercises prescribed by a physical therapist can help enhance the stability of the shoulder joint and reduce the risk of dislocations. Range of Motion Exercises: Incorporating exercises to improve shoulder flexibility and range of motion can be beneficial in maintaining joint health and stability. Muscle Balancing and Coordination Training: Working on muscle balance and coordination exercises can help ensure that all muscles around the shoulder joint are adequately strengthened and function optimally. Proper Technique and Form: When participating in sports or engaging in activities that involve the shoulder, it's important to use proper techniques and form to minimize the risk of injury. Avoiding Risky Movements: Individuals with a history of shoulder dislocations should be cautious and avoid activities or movements that are likely to cause a dislocation. Protective Gear: Wearing appropriate protective gear, such as shoulder braces or pads, during physical activities or sports can provide added support and reduce the risk of injury. Posture and Body Mechanics: Maintaining good posture and using proper body mechanics during activities can help reduce stress on the shoulder joint and decrease the risk of injury. Occupational Modification: Individuals with physically demanding occupations may need to modify their work tasks or use ergonomically designed tools to reduce strain on the shoulders. Follow Rehabilitation Plans: Adhering to rehabilitation plans and recommendations provided by healthcare professionals after a shoulder dislocation can support a strong and stable recovery. Consultation with a Specialist: If recurrent dislocations are a concern, consulting an orthopedic specialist for a thorough evaluation and discussion of potential surgical options for shoulder stabilization may be necessary.  Individuals with a history of shoulder dislocations should work closely with their healthcare team to develop a tailored prevention and rehabilitation plan. It's important to prioritize shoulder health and take proactive measures to minimize the risk of recurrent dislocations. |

**Supplemental Table D: Adhesive Capsulitis Questions and Answers**

| **Questions** | **Answers** |
| --- | --- |
| What is adhesive capsulitis (frozen shoulder)? | Adhesive capsulitis, commonly referred to as frozen shoulder, is a condition characterized by pain, stiffness, and limited range of motion in the shoulder joint. It typically affects the capsule, a connective tissue structure that surrounds the shoulder joint.  Here's a breakdown of what adhesive capsulitis (frozen shoulder) entails:  Pain and Discomfort: Individuals with adhesive capsulitis experience pain in the shoulder that may be dull or aching, often worsening at night. The pain may extend down the arm. Stiffness and Reduced Mobility: Stiffness in the shoulder is a hallmark of frozen shoulder. Range of motion is significantly limited, making it challenging to perform daily activities that involve shoulder movement.  Three Stages of Frozen Shoulder: Frozen shoulder typically progresses through three stages:  Freezing (Painful) Stage: Onset of pain and increasing stiffness. Frozen (Adhesive) Stage: Continued pain with severe stiffness and limited movement. Thawing Stage: Gradual improvement in pain and movement.  Gradual Development: The condition often develops slowly over time. Initially, people may notice discomfort and pain during certain movements. Over several months to years, the stiffness and limitations in movement can significantly worsen.  Causes and Risk Factors: The exact cause of frozen shoulder is often unknown, but it can be related to inflammation, scarring, or thickening of the capsule. Common risk factors include aging, being female, a history of shoulder injury or surgery, certain medical conditions (e.g., diabetes, thyroid disorders), and immobilization of the shoulder.  Diagnosis: Diagnosis is typically based on medical history, a physical examination, and imaging studies (X-rays, MRI) to rule out other potential shoulder issues and confirm the presence of adhesive capsulitis. Treatment: Treatment aims to relieve pain, improve range of motion, and restore shoulder function. It often involves a combination of physical therapy, stretching exercises, pain management with medications or injections, and in some cases, surgical intervention.  Frozen shoulder can significantly impact an individual's quality of life and daily activities. Early diagnosis and appropriate management are important to minimize discomfort and stiffness, and to facilitate a smoother recovery. It's important to work closely with a healthcare professional to develop an effective treatment plan based on the severity and stage of the frozen shoulder. |
| What causes adhesive capsulitis? | The exact cause of adhesive capsulitis, or frozen shoulder, is often not clearly understood. However, several factors and conditions are believed to contribute to its development. Adhesive capsulitis is characterized by inflammation, scarring, and thickening of the capsule surrounding the shoulder joint. Common factors and causes associated with adhesive capsulitis include:  Inflammation and Synovial Fluid Changes: Inflammation within the shoulder joint can cause irritation and thickening of the capsule. Changes in the composition of the synovial fluid (the lubricating fluid within the joint) may contribute to this inflammatory process. Lack of Use or Immobilization: Immobilization of the shoulder due to injury, surgery, or other reasons can lead to stiffness and a decrease in the normal range of motion. Lack of movement can cause the tissues in the shoulder to become less flexible and more prone to adhesive capsulitis. Scar Tissue Formation (Fibrosis): After an injury or inflammation, the body may produce excess scar tissue (fibrosis) in the shoulder joint, causing the capsule to thicken and contract, leading to stiffness and limited movement. Previous Shoulder Injuries or Surgeries: People who have had previous shoulder injuries, surgeries, or trauma to the shoulder may be at a higher risk of developing adhesive capsulitis. The trauma can trigger the inflammatory process and contribute to the development of the condition. Certain Medical Conditions: Adhesive capsulitis is more common in individuals with specific medical conditions, such as diabetes, thyroid disorders (hypothyroidism or hyperthyroidism), cardiovascular diseases, autoimmune disorders, and Parkinson's disease. Hormonal and Metabolic Factors: Hormonal changes or imbalances, particularly in women, may play a role in the development of frozen shoulder. Metabolic factors related to insulin resistance and glucose metabolism may also contribute. Genetic Predisposition: Some individuals may have a genetic predisposition to developing adhesive capsulitis. Family history can be a contributing factor. Age and Gender: Adhesive capsulitis is more common in individuals aged 40 to 60, and it tends to affect women more frequently than men.  Understanding the potential contributing factors can aid in the prevention, early detection, and appropriate management of adhesive capsulitis. However, individual experiences with the condition can vary, and it's important to consult a healthcare professional for a thorough evaluation and personalized treatment plan. |
| What are the symptoms of adhesive capsulitis? | Adhesive capsulitis, commonly known as frozen shoulder, is characterized by specific symptoms that can significantly affect the shoulder's mobility and cause varying degrees of pain and discomfort. The typical symptoms of adhesive capsulitis include:  Pain: Persistent and often severe pain in the shoulder, especially during movements or at rest, is a primary symptom. The pain can radiate down the arm. Stiffness: Gradual and progressive stiffness in the shoulder, limiting the ability to move the arm freely in different directions. Stiffness is a hallmark feature of frozen shoulder. Limited Range of Motion: Difficulty raising the arm, reaching overhead, or performing rotational movements due to the decreased range of motion in the shoulder joint. Difficulty Sleeping: Pain and discomfort may worsen at night, disrupting sleep and making it challenging to find a comfortable sleeping position. Painful Movements: Pain when attempting movements such as reaching behind the back, reaching for items on high shelves, or putting on/taking off clothes. Progressive Development: The symptoms usually develop gradually over time, progressing through three main stages: freezing stage, frozen stage, and thawing stage. Shoulder Weakness: Some individuals may experience weakness in the shoulder due to reduced use of the arm and muscle disuse atrophy. Painful Muscles: Muscles around the shoulder may feel tender or painful to the touch. Pain at Rest: Pain and discomfort may persist even when the shoulder is at rest. Difficulty with Activities of Daily Living: Difficulty performing routine activities like reaching for objects, brushing hair, putting on a seatbelt, or washing the back, due to the limited range of motion and pain.  It's important to note that these symptoms can vary in intensity and may fluctuate throughout the course of the condition. The duration of each stage and the severity of symptoms can also differ from person to person. If you suspect you have adhesive capsulitis or are experiencing persistent shoulder pain and stiffness, it's crucial to consult a healthcare professional for a thorough evaluation and appropriate management. Early diagnosis and intervention can help manage the symptoms and improve shoulder mobility. |
| How is adhesive capsulitis diagnosed and treated? | Diagnosing and treating adhesive capsulitis, or frozen shoulder, involves a combination of a thorough medical history review, physical examination, and, in some cases, imaging studies. Treatment aims to alleviate pain, improve shoulder mobility, and restore function. Here's a breakdown of the diagnostic process and common treatment approaches:  Diagnostic Process:  Medical History: The healthcare provider begins by taking a detailed history, asking about the onset and progression of symptoms, any previous injuries, medical conditions, or surgeries related to the shoulder. Physical Examination: A physical examination of the shoulder is conducted to assess pain, range of motion, strength, and any signs of inflammation or structural abnormalities. Imaging Studies: While not always necessary for diagnosis, imaging studies like X-rays, magnetic resonance imaging (MRI), or ultrasound may be used to rule out other potential shoulder conditions and assess the extent of the frozen shoulder.  Treatment Approaches:  Physical Therapy: Physical therapy is a cornerstone of treatment. A structured program involves exercises to improve shoulder mobility, stretching, strengthening, and range of motion exercises. The goal is to gradually restore the shoulder's function. Stretching Exercises: Gentle stretching exercises are crucial to improve shoulder flexibility and reduce stiffness. Patients are usually guided by a physical therapist to perform these stretches. Pain Management: Over-the-counter pain relievers (e.g., acetaminophen, NSAIDs) and, in some cases, prescription medications may be used to manage pain and discomfort associated with frozen shoulder. Corticosteroid Injections: Corticosteroid injections directly into the shoulder joint can provide temporary relief from pain and inflammation, facilitating improved mobility and participation in physical therapy. Hydrodilatation (Hydrosurgery): This procedure involves injecting a sterile fluid (usually a saline solution) into the shoulder joint to stretch and expand the capsule, promoting improved mobility. Manipulation Under Anesthesia (MUA): MUA is a procedure in which the shoulder is manipulated while the patient is under anesthesia, breaking up scar tissue and improving shoulder mobility. Surgical Intervention (Arthroscopic Release): In severe cases where conservative treatments haven't been effective, arthroscopic surgery may be considered. This involves releasing the tightened capsule and removing scar tissue to restore range of motion. Follow-Up Rehabilitation: Post-operative rehabilitation is crucial to regain shoulder strength and function, and it often involves an extended period of physical therapy and exercises.  Patient Education: Educating the patient about the condition, treatment options, and the importance of compliance with rehabilitation exercises is an integral part of the overall treatment plan.  Treatment plans are tailored to each individual's specific circumstances, considering factors such as the stage and severity of frozen shoulder, overall health, lifestyle, and goals. It's essential to work closely with a healthcare professional to determine the most suitable treatment approach and achieve the best outcome. |
| Can adhesive capsulitis be prevented or managed effectively? | While it's challenging to completely prevent adhesive capsulitis (frozen shoulder), there are strategies to reduce the risk of developing the condition or manage it effectively. Additionally, early intervention and appropriate management can help mitigate its impact and improve outcomes. Here are ways to potentially prevent and effectively manage adhesive capsulitis:  Prevention:  Regular Shoulder Exercise: Engage in regular shoulder exercises to maintain shoulder mobility and strength. This can help prevent stiffness and reduce the risk of developing frozen shoulder. Proper Posture: Maintain good posture to avoid unnecessary stress on the shoulder joints and muscles. Practice proper ergonomics in daily activities to reduce the risk of shoulder strain. Gradual Progression of Activities: Avoid abrupt or excessive increases in activity levels that may strain or stress the shoulder. Progress activities gradually to allow the shoulder to adapt. Early Treatment of Shoulder Injuries: Seek prompt medical attention and treatment for any shoulder injuries or conditions that may affect the shoulder joint, to prevent complications that could lead to adhesive capsulitis. Control Medical Conditions: If you have conditions like diabetes or thyroid disorders, work with your healthcare provider to manage and control them effectively, as these conditions may be associated with an increased risk of frozen shoulder.  Effective Management:  Early Diagnosis and Treatment: If you suspect the onset of frozen shoulder or experience shoulder pain and stiffness, consult a healthcare professional promptly for an accurate diagnosis and early intervention. Physical Therapy: Engage in a tailored physical therapy program with stretching and strengthening exercises designed to improve shoulder mobility, flexibility, and strength. Pain Management: Use prescribed pain medications and follow pain management techniques to alleviate discomfort associated with adhesive capsulitis. Regular Home Exercises: Complement in-clinic physical therapy with consistent home exercises as recommended by your healthcare provider. Adherence to a structured exercise regimen is crucial for improvement. Consistent Follow-Up: Attend scheduled follow-up appointments to monitor progress and adjust the treatment plan as needed. Patient Education: Learn about the condition, treatment options, and recommended exercises. Understand the importance of compliance and active participation in the recovery process.  Communication with Healthcare Providers: Maintain open communication with your healthcare team, reporting any changes or challenges in your condition to ensure appropriate adjustments to the treatment plan.  While complete prevention may not always be possible, early diagnosis and appropriate management can significantly improve outcomes and quality of life for individuals with adhesive capsulitis. It's essential to work closely with a healthcare professional for personalized guidance and treatment. |

**Supplemental Table E: Biceps Tendonitis Questions and Answers**

| **Questions** | **Answers** |
| --- | --- |
| What is biceps tendonitis, and what causes it? | Biceps tendonitis, also known as bicipital tendonitis, is a condition characterized by inflammation or irritation of the biceps tendon. The biceps tendon is a strong, cord-like structure that connects the biceps muscle in the upper arm to the shoulder and the forearm. Tendonitis refers to inflammation of the tendon, often accompanied by pain and discomfort in the affected area.  Here's a breakdown of what biceps tendonitis entails:  Anatomy: The biceps muscle has two heads (long head and short head) that join to form a single muscle belly. The tendon of the long head of the biceps runs from the shoulder joint and attaches to the top of the shoulder socket (glenoid) within the shoulder joint. Causes: Biceps tendonitis typically occurs due to repetitive overhead arm movements or repetitive use of the shoulder and arm. Common causes include: Overuse or Repetitive Strain: Continuous or repetitive overhead motions (e.g., throwing, lifting, reaching) can strain the biceps tendon, leading to inflammation and irritation. Age-Related Changes: As individuals age, wear and tear on the tendons can occur, making them more prone to inflammation and injury. Shoulder Impingement: When the space between the acromion (part of the shoulder blade) and the rotator cuff narrows, it can impinge on the biceps tendon, causing irritation and inflammation. Injury or Trauma: Direct injury to the shoulder, such as a fall or sudden impact, can damage the biceps tendon and result in tendonitis. Athletic Activities: Athletes involved in sports that require repetitive arm movements (e.g., baseball, tennis, weightlifting) are at higher risk of biceps tendonitis.  Symptoms: Symptoms of biceps tendonitis may include pain in the front of the shoulder or upper arm, especially during certain movements, such as reaching overhead or lifting. There may be tenderness, swelling, and localized warmth over the biceps tendon.  Diagnosis: Diagnosis typically involves a physical examination, medical history review, and sometimes imaging studies like X-rays, MRI, or ultrasound to visualize the condition of the tendon and rule out other potential shoulder issues.  Understanding the causes and symptoms of biceps tendonitis is crucial for appropriate management and treatment. Early intervention and proper care can help alleviate pain, reduce inflammation, and restore shoulder function. It's important to consult a healthcare professional for a thorough evaluation and personalized treatment plan. |
| What are the symptoms of biceps tendonitis? | Biceps tendonitis is characterized by specific symptoms primarily localized in the shoulder and upper arm area. Common symptoms of biceps tendonitis include:  Pain in the Front of the Shoulder: Persistent, dull, or sharp pain at the front of the shoulder, especially in the region where the biceps muscle inserts into the shoulder joint. Pain Radiating Down the Arm: Discomfort or pain that may radiate down the upper arm, sometimes reaching the elbow. The pain may worsen with certain movements. Pain During Activities: Pain exacerbated during activities that involve lifting, reaching, or overhead motions, such as lifting objects, throwing, or pushing. Tenderness and Swelling: Tenderness and localized swelling over the front of the shoulder, often concentrated near the biceps tendon attachment point. Weakness in the Arm: Reduced strength or weakness in the arm, particularly during activities that require the use of the biceps muscle. Crepitus: A crackling or grating sensation (crepitus) in the shoulder when moving the arm, which may be felt or heard. Difficulty with Specific Movements: Difficulty fully extending the arm or flexing the elbow due to pain or discomfort. Pain at Rest: Discomfort or pain even at rest, especially after activities that aggravate the biceps tendon. Limited Range of Motion: Reduced range of motion in the shoulder, particularly limitations in reaching overhead or behind the back. Warmth or Redness: In some cases, localized warmth or redness over the affected area may be observed.  It's important to note that the severity and duration of symptoms can vary from person to person. If you experience persistent or worsening symptoms indicative of biceps tendonitis, it's essential to seek medical evaluation and appropriate treatment. A healthcare professional can accurately diagnose the condition, recommend treatment options, and guide you in managing the symptoms effectively. |
| How is biceps tendonitis diagnosed? | The diagnosis of biceps tendonitis involves a combination of a thorough medical history review, a physical examination, and often imaging studies to confirm the condition and assess its severity. Here's how biceps tendonitis is typically diagnosed:  Medical History: The healthcare provider will begin by taking a detailed history, asking about the onset of symptoms, the specific location and nature of the pain, any previous injuries or activities that might have triggered the symptoms, and other relevant medical information. Physical Examination: A physical examination is essential to assess the shoulder, arm, and range of motion. The healthcare provider will palpate the shoulder to identify areas of tenderness and swelling and perform specific tests to evaluate biceps strength and function. Biceps Strength Testing: Testing the strength of the biceps muscle by having the individual perform various arm movements against resistance to evaluate muscle function. Range of Motion Assessment: Evaluating the range of motion of the shoulder and arm to determine any limitations or pain during specific movements, especially those that stress the biceps tendon. Provocative Tests: Specialized tests, such as the Speed's test or Yergason's test, may be performed to stress the biceps tendon and reproduce or exacerbate the symptoms.  Imaging Studies: While not always necessary for diagnosis, imaging studies may be used to confirm the presence of biceps tendonitis, rule out other potential issues, and assess the severity of the condition:  X-rays: These can help identify bone abnormalities, fractures, or arthritis. Magnetic Resonance Imaging (MRI): Provides detailed images of soft tissues, including the biceps tendon, to assess for inflammation, tears, or other abnormalities. Ultrasound: Real-time imaging that can evaluate the biceps tendon and surrounding structures, often used for a dynamic assessment of the tendon. Arthroscopy (in some cases): In more complex or severe cases, an arthroscopic examination of the shoulder joint may be performed to directly visualize the biceps tendon and surrounding structures.  By combining the medical history, physical examination, and imaging studies, healthcare professionals can accurately diagnose biceps tendonitis, determine the extent and severity of the condition, and develop an appropriate treatment plan tailored to the individual's needs. Early diagnosis is crucial for effective management and to prevent the worsening of symptoms. Always consult a healthcare provider for personalized evaluation and guidance. |
| What are the treatment options for biceps tendonitis? | The treatment for biceps tendonitis typically involves a combination of conservative approaches aimed at reducing pain, inflammation, and promoting healing of the biceps tendon. In more severe cases or if conservative treatments fail, surgical options may be considered. Here are the common treatment options for biceps tendonitis:  Rest and Activity Modification: Avoid or modify activities that aggravate the condition, particularly overhead movements or activities that strain the shoulder. Rest allows the tendon to heal and reduces stress on the affected area. Ice and Cold Therapy: Applying ice packs or cold compresses to the affected area can help reduce pain, inflammation, and swelling. Cold therapy can be applied for about 15-20 minutes, several times a day. Anti-Inflammatory Medications: Nonsteroidal anti-inflammatory drugs (NSAIDs), such as ibuprofen or naproxen, can help relieve pain and reduce inflammation in the tendon. Physical Therapy: A structured physical therapy program involving stretching and strengthening exercises can improve shoulder mobility, flexibility, and strength. Physical therapists can guide patients in performing appropriate exercises to aid in recovery. Steroid Injections: Corticosteroid injections directly into the affected area can provide short-term relief from pain and inflammation. However, these are typically limited to a few injections due to potential side effects. Ultrasound-Guided Needle Tenotomy (Tenex Procedure): This minimally invasive procedure involves using ultrasound guidance to insert a specialized tool into the tendon to remove damaged tissue and promote healing. Prolotherapy or Platelet-Rich Plasma (PRP) Injections: These treatments involve injecting a solution into the affected tendon to stimulate the body's natural healing process and improve tissue regeneration. Extracorporeal Shock Wave Therapy (ESWT): ESWT uses shock waves to stimulate blood flow and promote healing in the tendon, reducing pain and inflammation. Activity Modification and Ergonomic Changes: Adjusting movements and ergonomic habits to reduce strain on the affected shoulder can help in managing symptoms effectively. Surgical Intervention (in severe cases):  If conservative treatments are ineffective, surgical options may include:  Biceps Tenodesis: Surgically moving the biceps tendon to a different location to relieve pain. Biceps Tenotomy: Cutting and removing the damaged part of the biceps tendon to alleviate symptoms.  The choice of treatment depends on the severity of the condition, the individual's overall health, and their specific symptoms. Treatment plans are tailored to each individual's needs to achieve the best possible outcome. Always consult with a healthcare professional to determine the most appropriate treatment approach based on your situation. |
| Can biceps tendonitis be prevented, and what lifestyle modifications can help manage it? | While biceps tendonitis cannot be completely prevented, certain lifestyle modifications and proactive measures can help reduce the risk of developing this condition and manage its symptoms effectively. Here are some strategies for prevention and management:  Proper Warm-Up and Cool Down: Always perform adequate warm-up exercises before engaging in physical activities or sports that involve the shoulder. Gentle stretching and range of motion exercises can prepare the muscles and tendons for activity. Afterward, cool down with stretches to prevent stiffness and muscle tension. Strengthening Exercises: Incorporate regular strengthening exercises for the shoulder muscles, including the biceps, deltoids, and rotator cuff muscles. Strengthening these muscles can help provide stability to the shoulder joint and reduce the risk of overuse injuries. Progressive Training: Gradually increase the intensity, duration, and frequency of your training or exercise routines. Avoid sudden or extreme increases in activity, which can strain the tendons and muscles. Proper Technique and Form: Use correct form and technique during exercises, particularly those that involve the shoulder. Improper form can place unnecessary stress on the tendons and lead to injury. Avoid Overuse and Repetitive Movements: Limit repetitive overhead activities, heavy lifting, or excessive strain on the shoulder. If your work or activities involve repetitive motions, take regular breaks and vary your movements to prevent overuse. Ergonomic Workspace: Ensure that your workspace and equipment are ergonomically designed to support good posture and minimize strain on the shoulders and arms. Adjust chair height, desk setup, and computer monitor position accordingly. Maintain a Healthy Weight: Maintain a healthy body weight to reduce unnecessary stress on the joints, including the shoulder. Excess weight can strain the tendons and increase the risk of injury. Regular Stretching: Incorporate regular shoulder stretching exercises into your routine to improve flexibility and range of motion. Focus on the shoulder and biceps stretches to maintain mobility. Rest and Recovery: Ensure adequate rest and recovery time between workouts or physical activities to allow the muscles and tendons to heal and prevent overuse. Listen to Your Body: Pay attention to any early signs of discomfort or pain in the shoulder. If you experience pain during activities, stop and rest. Seek medical attention if pain persists. Regular Health Check-ups: Schedule regular check-ups with a healthcare professional to monitor your shoulder health and address any concerns promptly.  By incorporating these lifestyle modifications and practices, you can reduce the risk of developing biceps tendonitis and effectively manage its symptoms. Always consult with a healthcare professional for personalized advice and recommendations based on your specific circumstances. |
